# Association Between Breastmilk Microbiota and Food Allergy in Infants

**DOI:** 10.3389/fcimb.2021.770913

**Published:** 2022-01-12

**Authors:** Shuo Wang, Yuan Wei, Luyan Liu, Zailing Li

**Affiliations:** ^1^ Department of Pediatrics, Peking University Third Hospital, Beijing, China; ^2^ Department of Obstetrics and Gynecology, Peking University Third Hospital, Beijing, China

**Keywords:** breastmilk microbiome, breastfeeding, food allergies, butyrate, infant

## Abstract

Regulating the composition of human breastmilk has the potential to prevent allergic diseases early in life. The composition of breastmilk is complex, comprising varying levels of oligosaccharides, immunoactive molecules, vitamins, metabolites, and microbes. Although several studies have examined the relationship between different components of breastmilk and infant food allergies, few have investigated the relationship between microorganisms in breastmilk and infant food allergy. In the present study, we selected 135 healthy pregnant women and their full-term newborns from a cohort of 202 mother–infant pairs. Among them, 69 infants were exclusively breastfed until 6 mo after birth. At follow-up, 11 of the 69 infants developed a food allergy in infancy while 22 showed no signs of allergy. Thirty-three breastmilk samples were collected within 1 mo after delivery, and 123 infant fecal samples were collected at five time points following their birth. These samples were analyzed using microbial 16S rRNA gene sequencing. The abundance and evenness of the milk microbiota and the number of differential bacteria were higher in the breastmilk samples from the non-allergy group than in those from the food allergy group. The non-allergy group showed relatively high abundance of *Bifidobacterium*, *Akkermansia*, *Clostridium* IV, *Clostridium* XIVa, *Veillonella*, and butyrate-producing bacteria such as *Fusobacterium*, *Lachnospiraceae incertae sedis*, *Roseburia*, and *Ruminococcus*. In contrast, the abundance of *Proteobacteria*, *Acinetobacter*, and *Pseudomonas* in breastmilk was higher in the food allergy group. A comparison of the changes in dominant differential breastmilk microbiota in the intestinal flora of the two groups of infants over time revealed that the changes in *Bifidobacterium* abundance were consistent with those in the breastmilk flora. Functional pathway prediction of breastmilk microflora showed that the enhancement of the metabolic pathways of tyrosine, tryptophan, and fatty acids was significantly different between the groups. We suggest that changes in the breastmilk microbiota can influence the development of food allergies. Breastmilk contains several microbes that have protective effects against food allergies, both by influencing the colonization of intestinal microbiota and by producing butyrate. This study may provide new ideas for improving infant health through early intervention with probiotics.

## Introduction

Epidemiological studies have shown that the prevalence of food allergy is increasing worldwide, affecting up to 10% of the population (Lopes and Sicherer, 2020). Food allergy usually begins in the first 2 y of life (Sicherer and Sampson, 2014) and may have an impact on growth and lead to failure to thrive (Meyer, 2018). Severe food allergy may lead to anaphylaxis, which is life-threatening (Seth et al., 2020). Food allergies can also reduce the quality of life of children and their families (Meyer et al., 2017). Therefore, effective strategies to prevent food allergies have become a public health priority. Currently, breastfeeding is considered a pillar of primary allergy prevention (Heine, 2018), but its effectiveness remains controversial.

Human breast milk is considered the optimal source of nutrition for infants in their early life due to its ability to provide critical nutrients and bioactive compounds that support growth and immune development during infancy (Lyons et al., 2020). The World Health Organization recommends that infants are exclusively breastfed for the first six months of life (Kramer and Kakuma, 2012). The composition of breast milk is very complex; in addition to essential nutrients, bioactive ingredients (such as human milk oligosaccharides [HMOs], cytokines, chemokines, immunoglobulins, and hormones) and food antigens from the diet of mothers, breastmilk also contains a range of microorganisms that are important for maintaining the health of infants (Eriksen et al., 2018; Järvinen et al., 2019). To date, there have been several studies on the relationship between different components of breastmilk, such as food allergen active fragments (Rajani et al., 2020), immunoglobulins (Järvinen et al., 2014), and HMOs (Seppo et al., 2017; Miliku et al., 2018), and infant food allergies. However, there are only a few reports on the relationship between microorganisms in breastmilk and infant food allergy.

Breastmilk microbiota is currently thought to be associated with the development of childhood obesity (Oddi et al., 2020), childhood celiac disease (Benítez-Páez et al., 2020), pediatric asthma (Moossavi et al., 2018), and allergy development (Dzidic et al., 2020). Moreover, the effect of breastmilk microbiota on the health of offspring is considered to primarily occur *via* the colonization of intestinal flora (Järvinen et al., 2019). Growing evidence suggests that symbiotic microbiota play an important role in susceptibility to food allergies. Epidemiological studies have shown an association between exposure to known altered microbiota and the risk of food allergy (Bunyavanich and Berin, 2019). Breastmilk, as the only food in the early postnatal period for some infants, is an essential microbial source for developing infants (Pannaraj et al., 2017). Animal experiments have shown that the addition of *Lactobacillus* from breastmilk to the diet can reduce the occurrence and severity of milk protein allergic reactions in mice, suggesting that breastmilk flora may have a protective effect against food allergies (Lara-Villoslada et al., 2007). In this study, we aimed to establish a population cohort to observe the relationship between the microbiota of breastmilk and food allergy in infants by collecting and analyzing the microbiota in breastmilk and the intestinal microbiota of infants who were exclusively breastfed after birth.

## Materials and Methods

### Study Design

A nested case-control study was conducted in Peking University Third Hospital from February 2018 to May 2020. The institutional Ethics Committee of Peking University Third Hospital, Peking, China, approved this study protocol (Approval No. M2018022).

### Participants

Two hundred and two healthy pregnant women were recruited. Only pregnant women who had regular prenatal check-ups at our hospital and were deemed by the investigator to be in good physical and mental health conditions based on their medical history and examination were eligible to participate. The exclusion criteria included: 1) underlying diseases or pregnancy complications, 2) use of antibiotics or probiotics for 2 weeks before or after delivery, except prophylactic use (e.g., cesarean section), and 3) refusal to participate in the study. Written consent was obtained from all participants before their inclusion in the study. Subjects who met one or more of the following criteria during the course of the study were excluded from further participation: 1) premature birth (before 37 weeks), 2) postmature birth (after 42 weeks), 3) unstable vital signs after birth, 4) congenital malformation(s) in the infant, and 5) antibiotics or probiotics used in infants during the study.

### Determination of Food Allergy Manifestations

Infants were determined to have food allergies if they met the following conditions: 1) one or more manifestations of poor sleep, crying, anxiety, depression, rash, runny nose, sneezing, coughing, wheezing, vomiting, diarrhea, or blood in the stool; 2) food allergy symptoms disappeared or were ameliorated following avoidance of suspected allergenic food but returned or were aggravated after the reintroduction of the suspected food; and 3) positive result for allergen-specific immunoglobulin (Ig)E, food challenge test, or skin punctum test (one or more).

### Sample Collection

Breastmilk samples were collected within 1 mo after delivery and fecal samples of the neonates were collected at five time points (first defecation, 3 d after birth, and 1/3/6 mo after birth). Samples were collected by trained professionals during hospital or home visits and under a uniform protocol using sterile tubes with a DNA stabilizer (Sarstedt, Nümbrecht, Germany). All samples were thoroughly mixed and stored at −80°C within 6 h after collection until DNA extraction and sequencing.

### DNA Extraction, PCR Amplification, and High-Throughput Sequencing

DNA was extracted from each fecal sample using an improved protocol based on the QIAamp Fast DNA Stool Mini Kit (Qiagen, Hilden, Germany) manufacturer’s instructions. In detail, 1 mL of InhibitEX Buffer and glass beads (0.5 mm diameter, Qiagen) were added to each 200 mg feces sample. The mixture was homogenized and mixed at 60 Hz for 1 min twice with a homogenizer (FASTPREP-24, Aosheng Biotech, China). Subsequently, DNA purification was performed according to the manufacturer’s instructions.

The V3-V4 region of the bacteria 16S ribosomal RNA genes were amplified by PCR (95°C for 3 min, followed by 30 cycles at 98°C for 20 s, 58°C for 15 s, and 72°C for 20 s and a final extension at 72°C for 5 min) using barcoded primers 341F 5′-CCTACGGGRSGCAGCAG-3′ and 806R 5′-GGACTACVVGGGTATCTAATC-3′ (Wang and Qian, 2009). PCR reactions were performed in 30 μL mixtures containing 15 μL of 2 × KAPA Library Amplification ReadyMix, 1 μL of each primer (10 μM), 50 ng of template DNA and ddH2O (Pereira et al., 2017).

Amplicons were extracted from 2% agarose gels and purified using the AxyPrep DNA Gel Extraction Kit (Axygen Biosciences, Union City, CA, U.S.) according to the manufacturer’s instructions and quantified using Qubit^®^2.0 (Invitrogen, USA). All quantified amplicons were pooled to equalize concentrations for sequencing using Illumina MiSeq/HiSeq (Illumina, Inc., CA, USA). The paired end reads of 250 bp were overlapped at their 3′ ends for concatenation into original longer tags, using PANDAseq (https://github.com/neufeld/pandaseq, version 2.9). DNA extraction, library construction, and sequencing were conducted by Realbio Genomics Institute (Shanghai, China).

### 16S rRNA Gene Sequencing and Statistical Analysis

Assembled tags, trimmed of barcodes and primers, were further checked for their lengths and average base quality. 16S tags were restricted between 220 bp and 500 bp such that the average Phred score of bases was no lower than 20 (Q20) and no more than three ambiguous N. The copy number of tags was enumerated and redundancy of repeated tags was removed. Only the tags with a frequency > 1, which tend to be more reliable, were clustered into operational taxonomic units (OTUs). OTUs were clustered with 97% similarity using UPARSE (http://drive5.com/uparse/) and chimeric sequences were identified and removed using Usearch (version 7.0.1090) (Edgar, 2013). The representative OTU sequence was compared with that of the 16S database of known species (Ribosomal Database Project (RDP), http://rdp.cme.msu.edu) using the RDP method for classification (Cole et al., 2014).

Alpha diversity was evaluated using Chao1, Shannon, Simpson, and Observed_species indices, and the value of alpha diversity was calculated using QIIME (V1.9.1) software. Beta diversity was calculated using QIIME (V1.9.1) software and an iterative algorithm with weighted and unweighted species richness information. Gplots, vegan, and ade4 packages in R were used to analyze bacterial community composition and heatmap clustering at the genus level, multivariate ANOVA based on dissimilarities (Adonis), and principal coordinates analysis (PCoA), respectively. LEfSe software was used to perform linear discriminant analysis (LDA) effect size analysis (LEfSe) to identify potential microbial biomarkers (Segata et al., 2011). Using Phylogenetic Investigation of Communities by Reconstruction of Unobserved States (PICRUSt) v1.0.0 (Langille et al., 2013), functional predictions were made based on the enrichment of KEGG pathways from the 16S rRNA gene data. wilcox.test and kruskal.test in R package were separately used to analyze the difference between groups and among more than two groups, respectively.

### Analysis of General Characteristics of Participants

The general characteristics of the participants in the two groups were compared using Student’s *t*-test of normally distributed continuous variables, the Wilcoxon signed-rank test of R for non-normally distributed continuous variables, and the chi-square test for categorical variables.

## Results

### Participants

Of the 202 mother–infant pairs, 135 pairs, who met the criteria and were followed up regularly, were enrolled in the study following provision of signed informed consent by the mothers. Of these, 69 infants were exclusively breastfed until 6 mo after birth. The pairs were divided based on whether the infant had a food allergy at the age of 1 y; accordingly, 11 pairs were placed in the food allergy (FA) group and 22 in the non-allergy (NA) group (**Supplementary Figure 1**). The characteristics of mothers and infants are described in **Tables 1**, **2**, respectively. No significant differences were observed in the baseline characteristics between the groups. The incidence of food allergies was lower in exclusive breastfeeding infants than in non-exclusive breastfeeding infants, however, the difference was not significant (**Supplementary Table 1**).

**Table 1:** Characteristics of maternal participants.

Characteristic	Food allergy (n = 11)	Non-allergy (n = 22)	*P*
**Age (y)** ^a^	31.91 ± 2.63	32.74 ± 3.47	0.488
**Primigravida**			0.789
Yes, n (%)	8 (72.7)	15 (68.2)	
No, n (%)	3 (27.3)	7 (31.8)	
**Han ethnicity**			
Yes, n (%)	10 (90.9)	22 (100)	0.151
No, n (%)	1 (9.1)	0 (0)
**Employment**			
Yes, n (%)	11 (100)	21 (95.5)	0.473
No, n (%)	0 (0)	1 (4.5)
**Allergic symptoms**			
Yes, n (%)	6 (54.5)	10 (45.5)	0.622
No, n (%)	5 (45.5)	12 (54.5)

a Data are shown as mean ± standard deviation; p < 0.05 indicates a statistically significant difference.

**Table 2:** Characteristics of infant participants.

Characteristic	Food allergy (n = 11)	Non-allergy (n = 22)	*P*
**Gestational age at delivery (weeks) *^a^***	38.64 ± 0.92	38.57 ± 1.04	0.848
**Birth weight (g) *^a^***	3394.55 ± 448.83	3371.3 ± 253.51	0.848
**Height (cm) *^a^***	49.91 ± 1.87	50.17 ± 1.23	0.624
**Sex**			0.314
Male, n (%)	8 (72.7)	12 (54.5)	
Female, n (%)	3 (27.3)	10 (45.5)	
**Method of delivery**			
Vaginal delivery, n (%)	8 (72.7)	15 (68.2)	0.789
Cesarean, n (%)	3 (27.3)	7 (31.8)
**Use of antibiotics**			
Yes, n (%)	2 (18.2)	6 (27.3)	0.566
No, n (%)	9 (81.8)	16 (72.7)
**Contact with pets**			
Yes, n (%)	2 (18.2)	5 (22.7)	0.763
No, n (%)	9 (81.8)	17 (77.3)
**Time for complementary food**			
<6 mo, n (%)	3 (27.3)	8 (36.4)	0.602
>6 mo, n (%)	8 (72.7)	14 (63.6)
**Siblings**			
Yes, n (%)	3 (27.3)	9 (36.4)	0.443
No, n (%)	8 (72.7)	13 (63.6)

a Data are shown as mean ± standard deviation; p < 0.05 indicates a statistically significant difference.

### Reads and OTUs

**Figure 1:**
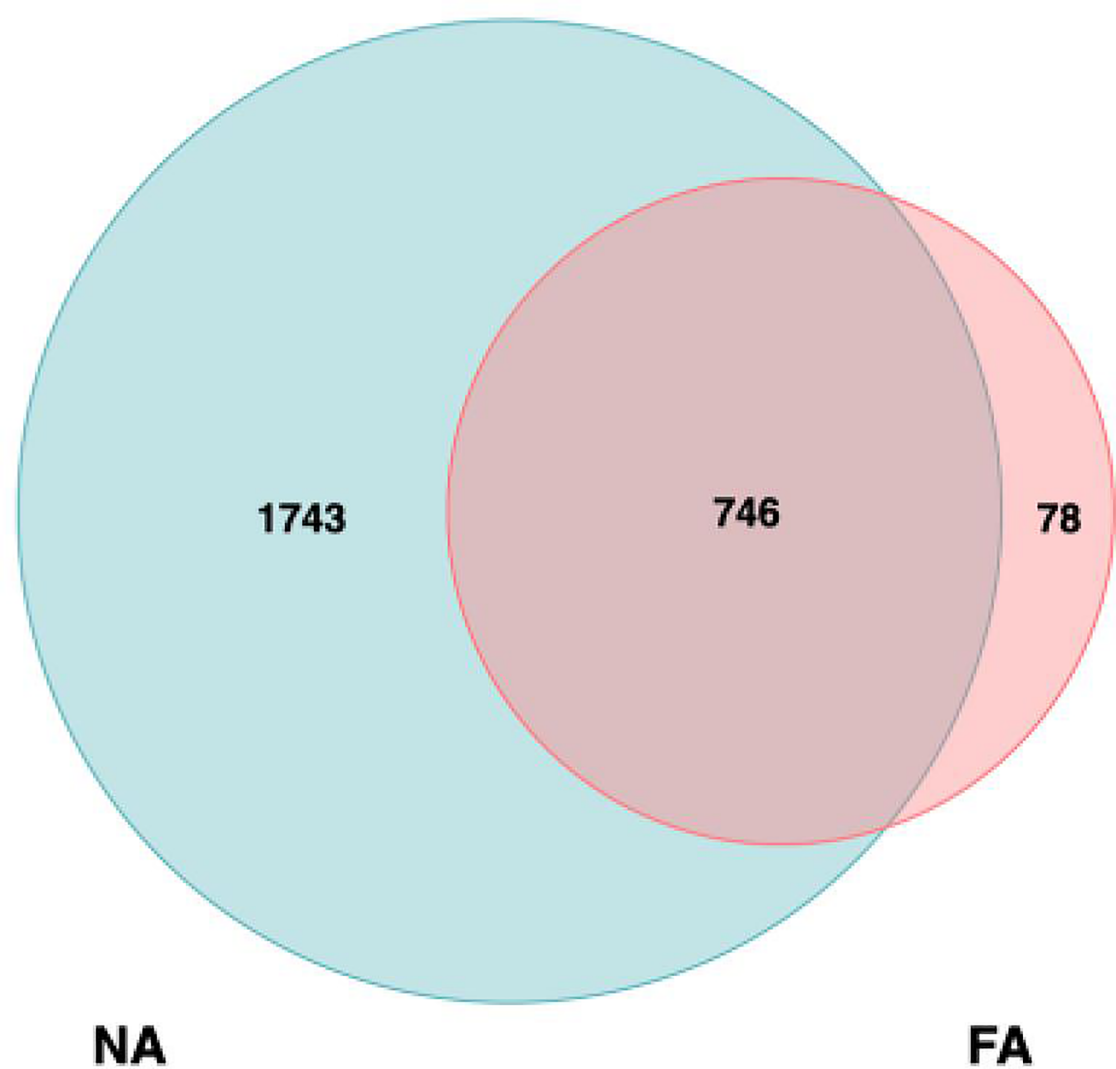
Analysis of operational taxonomic units (OTUs) in the non-allergy (NA) and food allergy (FA) groups. Numbers of unique and shared OTUs in the breastmilk microflora of the NA and FA groups.

We collected 33 breastmilk samples and 123 stool samples. Overall, 1,171,868 high-quality reads were obtained from the breastmilk samples, with 35,511 ± 1606 high-quality reads per sample, which were clustered into 2567 OTUs. Among these, 1743 and 78 were unique to the NA and FA groups, respectively, and 746 were shared between the groups (**Figure 1**).

### Comparison of Structures of Breastmilk Bacterial Communities Between the NA and FA Groups

**Figure 2:**
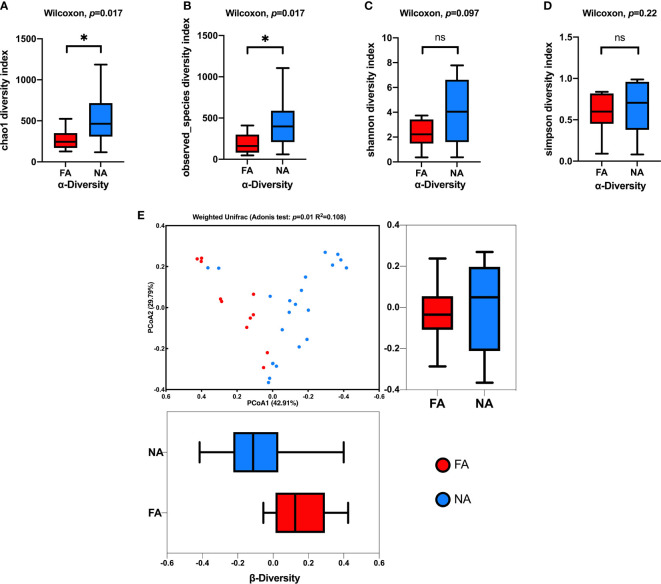
Comparison of the breastmilk microbial composition in the food allergy (FA) and non-allergy (NA) groups. **(A–D)** Alpha diversities of the FA and NA groups determined using the Chao1, Observed_species, Shannon, and Simpson indices, respectively. **(E)** Beta diversities of the FA and NA groups determined using the principal coordinates analysis (PCoA) combined with the Adonis analysis. ns, not significant. *p < 0.05.

The alpha and beta diversities of the breastmilk microbiota were compared between the FA and NA groups. We assessed alpha diversity between the groups using four indexes (**Figures 2A–D**). Chao1 and Observed_species indices reflect species richness, and Shannon or Simpson indices reflect species diversity. Compared with the FA group, the NA group showed high species richness and diversity, and Chao1 and Observed_species indices significantly differed between the groups.

We combined the PCoA with the Adonis analysis to evaluate the difference in beta diversity between the groups (**Figure 2E**). The PCoA results showed that the breastmilk microbiota of the two groups was separated into two clusters, and the Adonis analysis showed that the difference was significant (R^2^ = 0.108, *p* = 0.01).

### Comparison of Taxonomic Features of Breastmilk Microflora Between the NA and FA Groups

**Figure 3:**
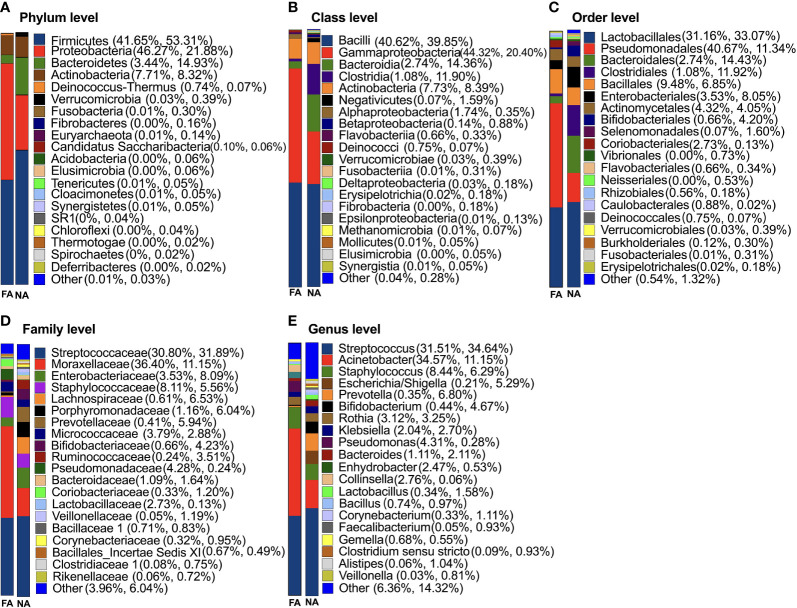
Classification of breastmilk microflora at the phylum **(A)**, class **(B)**, order **(C)**, family **(D)**, and genus **(E)** levels in the food allergy (FA) and non-allergy (NA) groups.

The results of classification and annotation at the phylum, class, order, family, and genus levels of all samples are shown in **Figure 3**. At the phylum level (**Figure 3A**), the predominant groups were, in the order of abundance, Firmicutes (41.65% and 53.31% of total bacteria in the FA and NA groups, respectively), Proteobacteria (46.27% in the FA group and 21.88% in the NA group), Bacteroidetes (3.44% in the FA group and 14.93% in the NA group), and Actinobacteria (7.71% in the FA group and 8.32% in the NA group). These four dominant phyla accounted for more than 95% of the bacteria in both groups. Compared with the NA group, the FA group had more Proteobacteria (*p* = 0.032) and less Bacteroidetes (*p* = 0.044) (**Table 3**).

**Table 3:** Comparison of breastmilk microbiota at the phylum level between the non-allergy and food allergy groups.

Phylum	Mean (FA)	Mean (NA)	*p* ^a^
p__Acidobacteria	3.67E-05	5.52E-04	0.017
p__Bacteroidetes	3.44E-02	1.49E-01	0.044
p__Chloroflexi	8.15E-06	3.57E-04	0.017
p__Elusimicrobia	1.22E-05	5.52E-04	0.036
p__Euryarchaeota	5.30E-05	1.39E-03	0.004
p__Fusobacteria	1.34E-04	3.01E-03	0.028
p__Proteobacteria	4.63E-01	2.19E-01	0.032
p__Tenericutes	6.11E-05	5.22E-04	0.033
p__Thermotogae	1.63E-05	2.22E-04	0.002
p__Verrucomicrobia	3.46E-04	3.91E-03	0.044

a p < 0.05 indicates a statistically significant difference.

At the genus level (**Figure 3E**), *Streptococcus* (31.51% and 34.64%) accounted for the highest proportion in the FA and NA groups, respectively, followed by *Acinetobacter* (34.57% and 11.15%) and *Staphylococcus* (8.44% and 6.29%). The remaining dominant bacteria (average relative abundance >1%) were as follows: *Escherichia*/*Shigella* (0.21% and 5.29%), *Prevotella* (0.35% and 6.80%), *Bifidobacterium* (0.44% and 4.67%), *Rothia* (3.12% and 3.25%), *Klebsiella* (2.04% and 2.70%), *Pseudomonas* (4.31% and 0.28%), *Bacteroides* (1.11% and 2.11%), *Enhydrobacter* (2.47% and 0.53%), *Collinsella* (2.76% and 0.06%), *Lactobacillus* (0.34% and 1.58%), *Bacillus* (0.74% and 0.97%), *Corynebacterium* (0.33% and 1.11%), *Gemella* (0.68% and 0.55%), *Clostridium sensu stricto* (0.09% and 0.93%), and *Alistipes* (0.06% and 1.04%). Although breastmilk contains abundant flora, differences exist in core genera among different studies on the composition and diversity of breastmilk, however, *Streptococcus* and *Staphylococcus* are consistently reported as core genera (Jost et al., 2013; Murphy et al., 2017; Chen et al., 2018), which agrees with the findings of our study.

### Differences in the Relative Abundance of Breastmilk Bacteria Between the Groups

**Figure 4:**
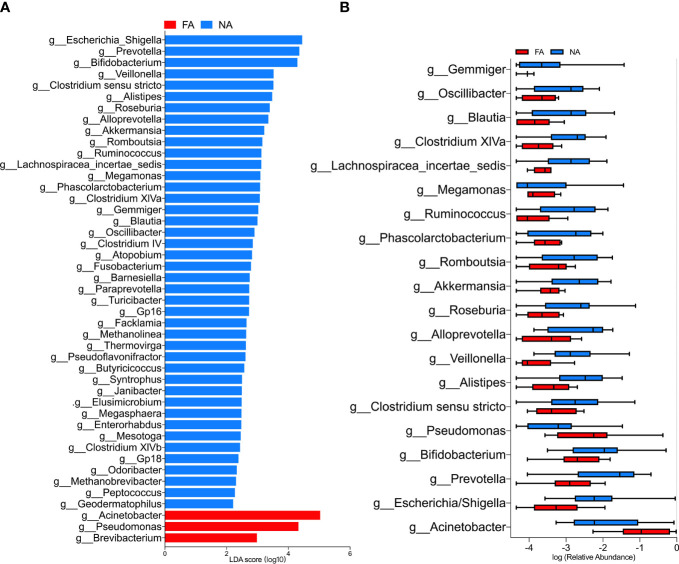
**(A)** LEfSe analysis between the food allergy (FA) and non-allergy (NA) groups, showing genera with absolute values of linear discriminant analysis (LDA) score > 2.0. **(B)** Top 20 genera with significantly different abundances between the FA and NA groups.

The LEfSe analysis (Segata et al., 2011) was performed to explore the relative abundance of taxa, characterized by significant differences between the groups. Among the 46 genera with different relative abundances (**Table 4**), 45 had the absolute value of log_10_ (LDA score) > 2.0 (**Figure 4A**). The top 20 genera are shown in **Figure 4B**. The NA group included more differential genera than the FA group. In particular, the FA group had a higher abundance of *Acinetobacter* (*p* = 0.006) and *Pseudomonas* (*p* = 0.019) and a lower abundance of *Escherichia*/*Shigella* (*p* = 0.011), *Prevotella* (*p* = 0.007), *Bifidobacterium* (*p* = 0.034), *Clostridium sensu stricto* (*p* = 0.023), *Veillonella* (*p* = 0.001), *Roseburia* (*p* = 0.037), *Akkermansia* (*p* = 0.033), *Ruminococcus* (*p* = 0.015), *Lachnospiracea incertae sedis* (*p* = 0.005), *Clostridium* XlVa (*p* = 0.010), and *Blautia* (*p* = 0.017). These genera might represent candidates for microbial biomarkers between the two groups. In fact, *Bifidobacterium* has been used as a probiotic in food allergies (Mennini et al., 2021). In addition, microorganisms including *Veillonella*, *Clostridium sensu stricto*, and *Akkermansia* reportedly have protective effects on host allergic diseases in intestinal flora studies (Tanaka et al., 2017; Shen et al., 2019).

**Table 4:** Comparison of breastmilk microbiota at the genus level between the non-allergy and food allergy groups.

Genus	Mean (FA)	Mean (NA)	*p* ^a^
g__*Acinetobacter*	3.40E-01	1.03E-01	0.006
g__*Prevotella*	3.39E-03	4.86E-02	0.007
g__*Escherichia/Shigella*	2.05E-03	4.96E-02	0.011
g__*Pseudomonas*	4.28E-02	2.33E-03	0.019
g__*Bifidobacterium*	4.25E-03	4.07E-02	0.034
g__*Clostridium sensu stricto*	8.39E-04	7.29E-03	0.023
g__*Alistipes*	6.11E-04	6.83E-03	0.01
g__*Veillonella*	2.73E-04	6.65E-03	0.001
g__*Alloprevotella*	6.36E-04	5.53E-03	0.019
g__*Roseburia*	3.10E-04	5.54E-03	0.037
g__*Romboutsia*	4.85E-04	3.60E-03	0.017
g__*Akkermansia*	3.14E-04	3.74E-03	0.033
g__*Phascolarctobacterium*	2.16E-04	2.80E-03	0.011
g__*Ruminococcus*	1.79E-04	2.81E-03	0.015
g__*Lachnospiracea*_*incertae_sedis*	1.55E-04	2.78E-03	0.005
g__*Megamonas*	9.78E-05	2.82E-03	0.024
g__*Clostridium* XlVa	2.24E-04	2.68E-03	0.01
g__*Blautia*	2.32E-04	2.53E-03	0.017
g__*Oscillibacter*	2.20E-04	1.91E-03	0.03
g__*Gemmiger*	2.44E-05	1.97E-03	0.009
g__*Barnesiella*	2.93E-04	1.49E-03	0.037
g__*Clostridium* IV	1.71E-04	1.61E-03	0.007
g__*Fusobacterium*	8.96E-05	1.57E-03	0.045
g__*Turicibacter*	6.52E-05	1.23E-03	0.008
g__*Pseudoflavonifractor*	6.52E-05	8.62E-04	0.045
g__*Paraprevotella*	8.15E-06	8.27E-04	0.036
g__*Megasphaera*	5.30E-05	5.50E-04	0.033
g__*Clostridium* XlVb	6.11E-05	5.20E-04	0.027
g__*Elusimicrobium*	1.22E-05	5.34E-04	0.036
g__*Butyricicoccus*	3.67E-05	4.95E-04	0.027
g__*Odoribacter*	6.11E-05	4.69E-04	0.017
g__*Flavonifractor*	3.26E-05	3.95E-04	0.04
g__*Thermovirga*	3.26E-05	3.57E-04	0.028
g__*Methanobrevibacter*	0	2.93E-04	0.027
g__Gp18	1.63E-05	2.24E-04	0.034
g__*Enterorhabdus*	1.63E-05	2.02E-04	0.045
g__*Mesotoga*	1.63E-05	1.81E-04	0.005
g__*Peptococcus*	0	1.47E-04	0.01
g__*Geodermatophilus*	4.07E-06	1.22E-04	0.049
g__*Atopobium*	0	1.08E-04	0.017
g__*Facklamia*	0	8.35E-05	0.027
g__*Methanolinea*	0	8.35E-05	0.042
g__Gp16	0	6.93E-05	0.027
g__*Syntrophus*	0	6.93E-05	0.042
g__*Janibacter*	0	5.70E-05	0.042
g__*Brevibacterium*	2.04E-05	0	0.047

a p < 0.05 indicates a statistically significant difference.

### Variations in the Fecal Microbiota of Infants Over Time in the NA and FA Groups

**Figure 5:**
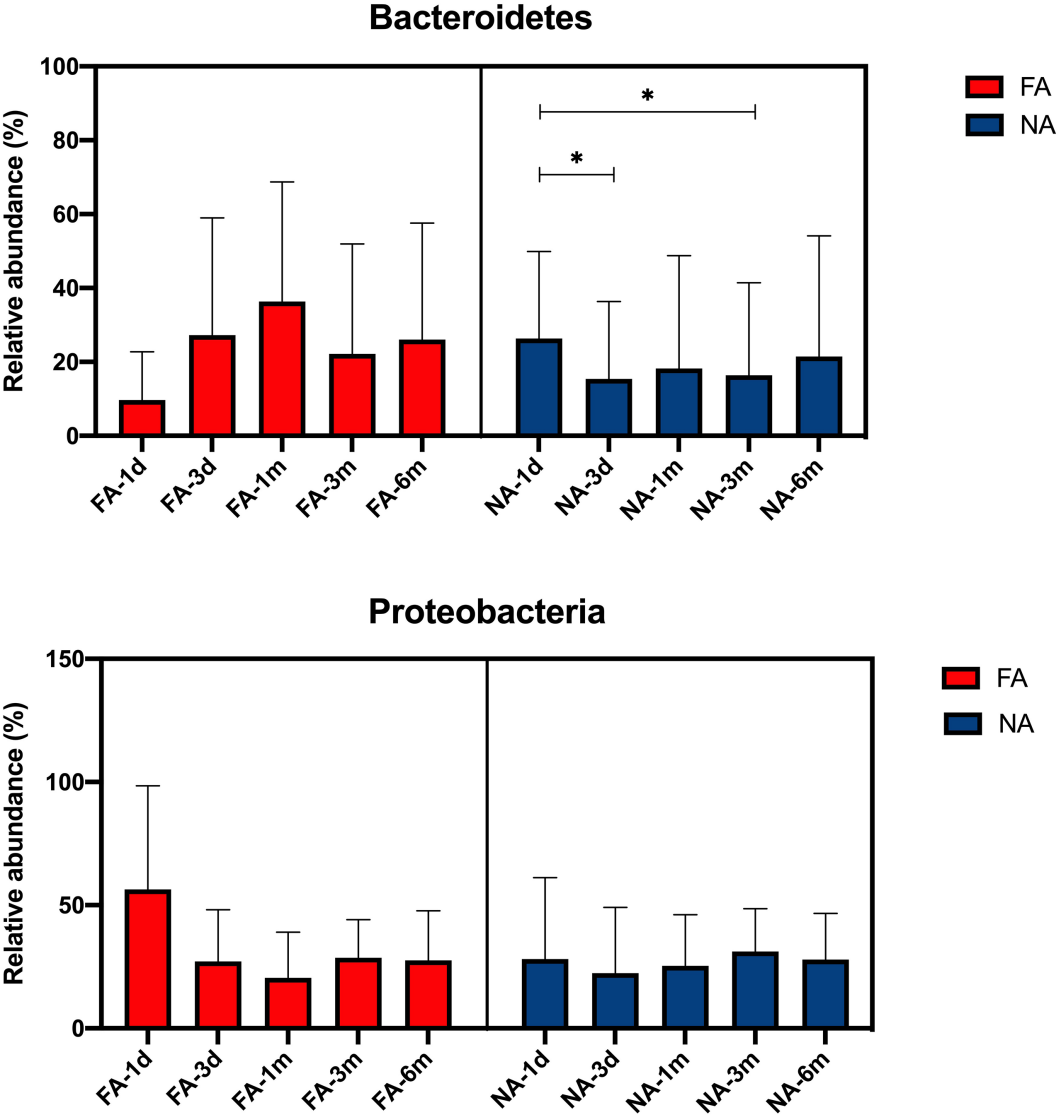
Main differential breastmilk microflora at the phylum level in the intestinal microflora of infants over time in the food allergy (FA) and non-allergy (NA) groups. *p < 0.05.

We further compared the changes in the dominant breastmilk microbiota, which showed a significant difference between the groups (average relative abundance > 1%) in the intestinal tract of infants over time. At the phylum level, the dominant bacteria included Bacteroidetes and Proteobacteria, and the changing trend of their relative abundance time is shown in **Figure 5**. In the FA group, the relative abundance of Bacteroidetes gradually increased within 1 mo after birth (36.3%), decreased at 3 mo (22.2%), and increased again at 6 mo (26.1%). In the NA group, the relative of Bacteroidetes was the highest at 26.3% at 1 d (meconium), then decreased to 15.4% at 3 d, increased to 18.2% at 1 mo, and decreased to 16.4% at 3 mo, and increased to 21.5% at 6 mo. During 3 d to 6 mo, the relative abundance of Bacteroidetes in the NA group was lower than that in the FA group. Meanwhile, the relative abundance of Proteobacteria in the FA group decreased gradually within 1 mo after birth, reaching 56.4% at 1 d (meconium), significantly decreasing to 27.1% at 3 d, decreasing to the lowest level of 20.5% at 1 mo, increasing to 28.7% at 3 mo, and decreasing to 27.6% at 6 mo. Furthermore, in the NA group, the relative abundance of Proteobacteria fluctuated in a small range after birth, reaching 28.1% at 1 d (meconium), a slight decrease to 22.4% at 3 d, a gradual increase to 25.4% at 1 mo, a further increase to a peak of 31.2% at 3 mo, and a decrease to 27.9% at 6 mo, which was close to the level observed at 6 mo in the FA group.

**Figure 6:**
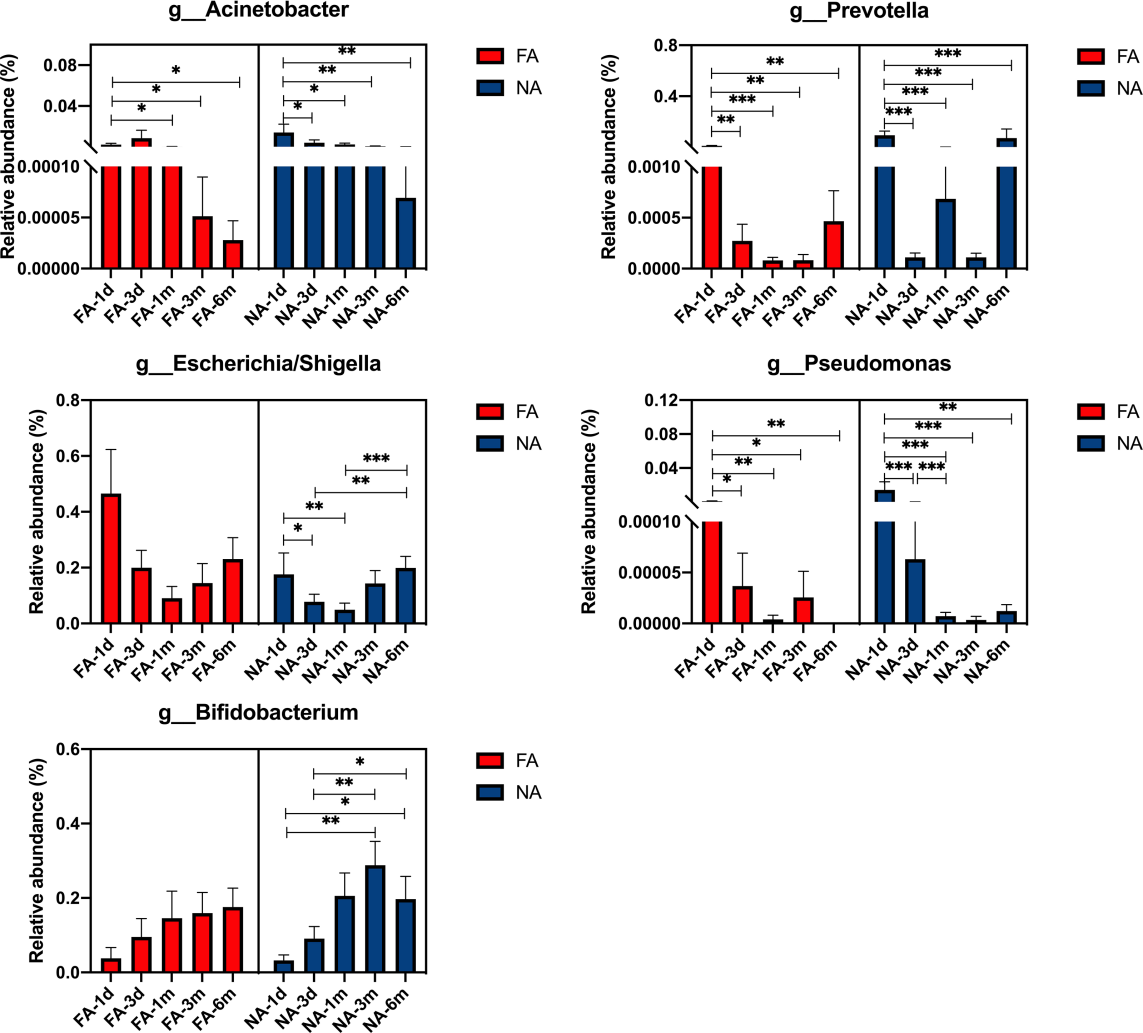
Main differential breastmilk microflora at the genus level in the intestinal microflora of infants over time in the food allergy (FA) and non-allergy (NA) groups. *p < 0.05, **p < 0.01, ***p < 0.001.

At the genus level, the dominant bacteria included *Acinetobacter*, *Prevotella*, *Escherichia*/*Shigella*, *Pseudomonas*, *Bifidobacterium.* The relative abundance of each genus increased and decreased after birth (**Figure 6**). The relative abundance of *Acinetobacter* was the highest at 3 d (0.86%) in the FA group and at 1 d (1.41%) in the NA group. After 3 d, the relative abundance of *Acinetobacter* decreased gradually in both groups, especially in the FA group. The relative abundance of *Prevotella* in both groups was the highest at 1 d (FA 1.20%, NA 9.42%) and decreased at 3 d (FA 0.03%, NA 0.01%). The relative abundance of *Prevotella* decreased to the lowest level of 0.0% at 1 mo in the FA group, whereas in the NA group, it increased to 0.07% at 1 mo. The level in both groups was lower at 3 mo (FA 0.00%, NA 0.01%), and increased at 6 mo (FA 0.05%, NA 7.15%). The relative abundance of *Escherichia/Shigella* showed a similar trend over time in the two groups, with a relatively high level at 1 d (FA 46.52%, NA 17.57%), gradually decreased to the lowest level at 1 mo (FA 9.08%, NA 4.94%), and then increased gradually to 23.06% in the FA group and 19.90% in the NA group. Within 6 mo after birth, the relative abundance of *Escherichia/Shigella* in the NA group was lower than that in the FA group. The relative abundance of *Bifidobacterium* showed a gradually increasing trend after birth. It was lower at 1 d (FA 3.81%, NA 3.22%), and slightly increased at 3 d (FA 9.53%, NA 9.07%). It continued to increase at 1 and 3 mo (1 mo: FA 14.58%, NA 20.55%; 3 mo: FA 15.93%, NA 28.79%), although there was a slight decrease in the NA group at 6 mo, it was still higher than that in the FA group (FA 17.57%, NA 19.69%). The relative abundance of *Pseudomonas* was the highest at 1 d (FA 0.08%, NA 1.43%), and then decreased gradually. Meanwhile, after 3 d, the relative abundance of *Pseudomonas* was very low (average relative abundance < 0.01%). Although these microbiota represent dominant differential species in breastmilk of the FA and NA groups, their relative abundance in the gut microbiota of exclusively breastfed infants did not differ significantly between the two groups at the same time point. With the exception of *Bifidobacterium*, the relative abundance of other bacteria in the intestinal microbiota did not gradually increase with time, but rather fluctuated over time.

### Predicted Functional Pathways of Breastmilk Bacterial Taxa Associated With Food Allergies

**Figure 7:**
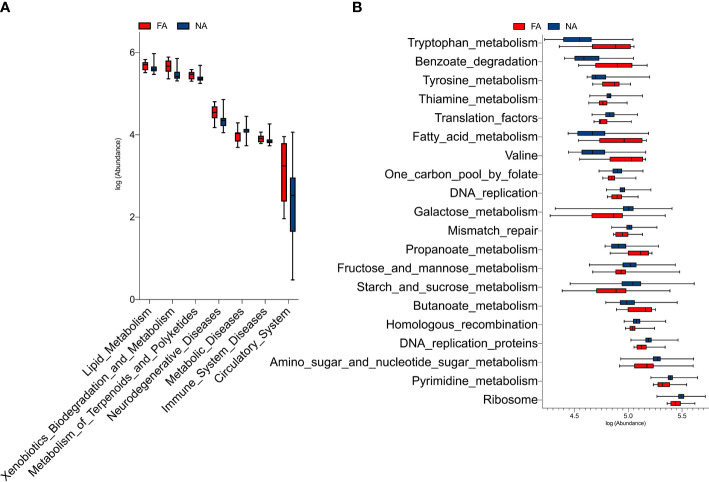
KEGG pathways at level 2 **(A)** and level 3 **(B)** with significantly different abundances between the food allergy (FA) and non-allergy (NA) groups.

The PICRUSt analysis revealed that seven KEGG pathways at level 2 significantly differed in abundance between the FA and NA groups (**Figure 7A**). Genes in the FA group seemed to be overrepresented in pathways related to metabolism, human diseases, and organismal systems (*p* < 0.05). The top 20 KEGG pathways at level 3 that differed significantly in abundance between the groups are shown in **Figure 7B**. The FA group had a higher level of butanoate and propanoate metabolism (*p* = 0.019 and *p* = 0.003, respectively); valine, leucine, and isoleucine degradation (*p* = 0.002); fatty acid metabolism (*p* = 0.006); tyrosine and tryptophan metabolism (*p* = 0.004 and *p* = 0.004, respectively); and benzoate degradation (*p* = 0.002). Hence, these pathways could possibly play important roles in the development of food allergies.

## Discussion

Breastmilk is rich in various components, and microbiota is one of them. Breastmilk is considered the best source of nutrition for infants during the first 6 mo of life, and therefore, it is necessary to study the effects of microbiota in breastmilk on the health of infants. Therefore, our study aimed to describe the relationship between breastmilk microbiota and FA in infants by establishing a small mother–infant cohort. We selected pairs in which the infants were exclusively breastfed within 6 mo of birth to avoid interference from formula intake. We also compared some influencing factors that may interfere with the microflora of breastmilk, such as maternal age, gestational age, parity, race, and delivery method (Moossavi et al., 2019; Zimmermann and Curtis, 2020); we observed no differences in these influencing factors between the FA and NA groups. Therefore, our cohort study provides a good description of the relationship between breastmilk microbiota and food allergies in infants.

Recent studies have shown that breastmilk provides a quarter of the intestinal microbiota of infants (Pannaraj et al., 2017), and current studies on the relationship between breastmilk flora and allergy primarily considered that breastmilk microbiota works by influencing the gut bacteria colonize (Moossavi et al., 2018; Järvinen et al., 2019). We, therefore, also sought to compare the primary differences in breastmilk microflora dynamics in the infant intestinal microbiota in the cohort. To this end, we collected and analyzed the intestinal microbiota of exclusive breastfeeding infants at 1 d, 3 d, 1 mo, 3 mo, and 6 mo after birth to observe the changes of dominant breastmilk microbiota in the intestinal microbiota of infants over time, and to further investigate the role of breastmilk microbiota in FA.

The breastmilk microbiota of the NA group had more differentiated bacteria from the genera *Akkermansia*, *Bifidobacterium*, *Clostridium* IV, *Clostridium* XIVa, and *Veillonella* than the FA group. This is consistent with the findings of several studies on the relationship between intestinal microbiota and food allergies in infants (Tanaka et al., 2017; Shen et al., 2019) and oral microbiota (Ho et al., 2021). Among them, *Bifidobacterium* has been widely recognized in the prevention of food allergies and has a long history of safe use (Enomoto et al., 2014; Tanaka et al., 2017; Liu et al., 2018); *Clostridium* IV has also demonstrated a protective effect against food allergies in animal experiments (Kowalczyk et al., 2021). *Clostridium* IV and *Clostridium* XIVa promote T(reg) cell accumulation and increase resistance to systemic immunoglobulin E responses in mouse models (Atarashi et al., 2011).

We also found that the relative abundance of *Prevotella* in the breastmilk microbiota was significantly higher in the NA group than in the FA group. Although previous studies have reported that the abundance of *Prevotella* is increased in the intestinal microbiota of infants with cow’s milk allergy (CMA) (Mennini et al., 2021), other studies have reported that a high abundance of *Prevotella* in the maternal intestinal microbiota during pregnancy has a protective effect against allergic diseases in infants. However, this protective effect was not associated with the relative abundance of *Prevotella* in the infant’s intestinal microbiota, suggesting that certain metabolites of *Prevotella* may have a protective effect against allergic diseases (Vuillermin et al., 2020). In our study, no difference was observed in the relative abundance of *Prevotella* in the intestinal microbiota of infants between the two groups, suggesting that it does not affect FA by altering the colonization of intestinal microbiota, however, the specific mechanism requires further analysis.

Our study showed that the abundance of some butyrate-producing bacteria, such as *Fusobacterium*, *Lachnospiraceae incertae sedis*, *Roseburia*, and *Ruminococcus*, was significantly increased in the NA group compared with that in the FA group. Recent studies have proposed that as a key component of breastmilk, butyrate can prevent the occurrence of food allergies (Paparo et al., 2020). Thus, it can be further demonstrated that breastmilk microflora is likely to prevent the occurrence of FA in infants by producing butyrate.

However, some pathogenic bacteria were also relatively abundant in the breastmilk of the NA group. For example, *Escherichia*/*Shigella* is considered one of the top pathogenic bacteria causing moderate to severe diarrhea in African and South Asian children (Kotloff et al., 2013). In the intestinal microbiota of infants in this study, the relative abundance of pathogenic bacteria in the FA group was higher than that in the NA group, suggesting that *Escherichia*/*Shigella* in breastmilk did not affect bacterial colonization in the intestinal microbiota.

In the FA group, the relative abundance of *Proteobacteria*, *Acinetobacter*, and *Pseudomonas* in breastmilk microbiota was significantly higher than that in the NA group, suggesting that they are related to the development of FA. Previous studies have found that the relative abundance of *Acinetobacter* in the intestinal microbiota of infants with allergic diseases is higher than that in the healthy group (Shen et al., 2019), which is consistent with the results of our study. *Pseudomonas* is generally considered pathogenic. Moreover, a recent study also found that the relative abundance of *Pseudomonas* in the breastmilk of infants with allergic symptoms in childhood was significantly increased in the first month after birth (Dzidic et al., 2020).

Among the intestinal flora, the relative microbial abundance in the meconium is likely most reflective of the intrauterine fetal intestinal microbiota and is not closely related to breastmilk microbiota, however, may serve as a reference. In the present study, at the phylum level, the relative abundance of Proteobacteria and Bacteroidetes fluctuated over time with no significant difference observed between the two groups at each time point, which was consistent with another study on intestinal microbiota and allergy diseases (Shen et al., 2019). At the genus level, the relative abundance of *Bifidobacterium* increased gradually over time in both groups, which may be related to the fact that human milk HMO can stimulate the growth of *Bifidobacterium* in the intestinal microbiota of infants (Vandenplas et al., 2020). However, the relative abundance of *Bifidobacterium* in the NA group was higher than that in the FA group. We further speculated that *Bifidobacterium* could also directly affect the colonization of intestinal microbiota through breastmilk, thus playing a role in preventing food allergies. Moreover, the relative abundance of *Acinetobacter* decreased gradually over time in both groups, however, exhibited a more marked, yet insignificant, decrease in in the FA group than the NA group, which was inconsistent with the gradual colonization of breastmilk microbiota in intestinal microbiota. Although *Pseudomonas* was dominant in the breastmilk differential microbiota, its relative abundance in the intestinal microbiota of infants after birth was very low in both groups. Our results show that the composition of the breastmilk microbiota is not completely consistent with the composition and changes of the intestinal microbiota of exclusive breastfeeding infants, which may be related to the influence of multiple factors in the process of changing the intestinal microbiota over time, such as the mode of delivery mode, the application of antibiotics, the addition of complementary food, the living environment, siblings, and sex (Chong et al., 2018; Kim et al., 2019). We compared the related factors affecting the changes in intestinal microbiota in the two groups of infants and found no significant difference between the groups (**Table 2**). This also indicated that breastmilk microbiota can affect intestinal microbiota to some extent, but not completely through the intestinal microbiota to affect FA.

Significant differences were identified in tryptophan, tyrosine, and fatty acid metabolism between the FA and NA groups in our study, suggesting that microflora may play a role in food allergy through these metabolic pathways. A previous study that compared serum samples from infants with food allergies with those without atopic disorders found significant differences in fatty acid metabolism between the groups (Crestani et al., 2020). Another study found that increased tyrosine metabolism was associated with food allergies (Chiu et al., 2020); however, the mechanism remains unclear. Tryptophan metabolism has also been associated with asthma (Checkley et al., 2016; Carraro et al., 2018). In a non-metabolomic study of 205 children, the levels of tryptophan and its downstream metabolite kynurenine were significantly higher in children with allergic diseases, such as asthma, rhinitis, and atopic dermatitis, than in healthy control children (Ünüvar et al., 2019). However, evidence linking tryptophan metabolism to FA is limited.

By establishing a cohort, we demonstrated that the differences in breastmilk microbiota, present before the onset of FA symptoms, are associated with the development of food allergies in infants. However, certain limitations of the study need to be acknowledged. The components of breastmilk are rich and complex. At present, it is known that immunoglobulins (Rajani et al., 2020) and HMOs (Järvinen et al., 2014; Seppo et al., 2017) in breastmilk have protective effects against allergic diseases of infants, whereas we assumed that the components of breastmilk are uniform, except for bacteria. Various components of breastmilk interact with each other, but it is undeniable that the microflora is an important component, and it is worth emphasizing that our study is currently the only study on the relationship between breastmilk microbiota and FA in infants.

The results of our study indicate that breastmilk contains an abundant microbiota, which is not identical in each individual due to the influence of multiple factors, and these differences have an association with the occurrence of food allergies in infants later in life. Even exclusive breastfeeding infants cannot be completely protected from food allergies, but it is clear that breastmilk contains many bacteria associated with FA prevention that can influence the development of food allergies by affecting gut microflora colonization, but not entirely by changing gut microflora colonization. These breastmilk microbiota associated with FA prevention contain both proven probiotics and many unproven differential bacteria, and further *in vitro* studies are needed to assess their potential as probiotics to ensure their safety and efficacy in humans. If proven to be probiotics, the microbiome could be improved by adding probiotics to breastmilk or milk formula, providing a viable avenue for early intervention to improve infant health in the future. Breastmilk also contains some bacteria associated with food allergies, and their mechanisms of action need to be further studied.

## Conclusions

In this study, exclusively breastfed infants were selected to compare the microbiota in the breast milk consumed by infants with food allergies and infants without allergies. We found that breastmilk is rich in microflora, and a high abundance and evenness of the flora has a protective effect on the occurrence of food allergies in infants. Breastmilk contains various beneficial bacteria, including *Bifidobacterium*, *Akkermansia*, *Clostridium* IV, *Clostridium* XlVa, and *Veillonella*, and some butyrate-producing bacteria, such as *Fusobacterium*, *Lachnospiracea incertae sedis*, *Roseburia*, and *Ruminococcus*, which have been linked to the prevention of food allergies in infants. *Bifidobacterium* in breastmilk probably influence the development of food allergies through intestinal colonization. Beneficial bacteria in breastmilk possibly prevent food sensitivity by producing butyrate, but the exact mechanism needs to be further studied. Breastmilk microbiota perhaps also influence the development of food allergies in infants by affecting tryptophan, tyrosine, and fatty acid metabolism. Breastmilk also contains pathogenic bacteria, such as *Escherichia/Shigella*, however, does not affect its colonization in the intestinal microbiota.

## Data Availability Statement

The original contributions presented in the study are publicly available in NCBI under accession number PRJNA766335.

## Ethics Statement

The studies involving human participants were reviewed and approved by The Institutional Ethics Committee of Peking University Third Hospital. Written informed consent to participate in this study was provided by the participants’ legal guardian/next of kin.

## Author Contributions

ZL and SW conceived this study. SW, YW, LL, and ZL recruited participants and collected samples. XC and PW provided sequencing technical assistance. SW wrote the manuscript and prepared the figures. All authors provided critical intellectual content and approved the final manuscript.

## Conflict of Interest

The authors declare that the research was conducted in the absence of any commercial or financial relationships that could be construed as a potential conflict of interest.

## Publisher’s Note

All claims expressed in this article are solely those of the authors and do not necessarily represent those of their affiliated organizations, or those of the publisher, the editors and the reviewers. Any product that may be evaluated in this article, or claim that may be made by its manufacturer, is not guaranteed or endorsed by the publisher.
