# Supplementary material for: Association Between Breastmilk Microbiota and Food Allergy in Infants
Source: Front Cell Infect Microbiol. 2022 Jan 12;11:770913. doi: 10.3389/fcimb.2021.770913 (PMC8790183; doi:10.3389/fcimb.2021.770913)
Supplement: Supplementary file 1 [file Table_1.docx]

Supplementary Material

# Supplementary Figures and Tables

## Supplementary Figure


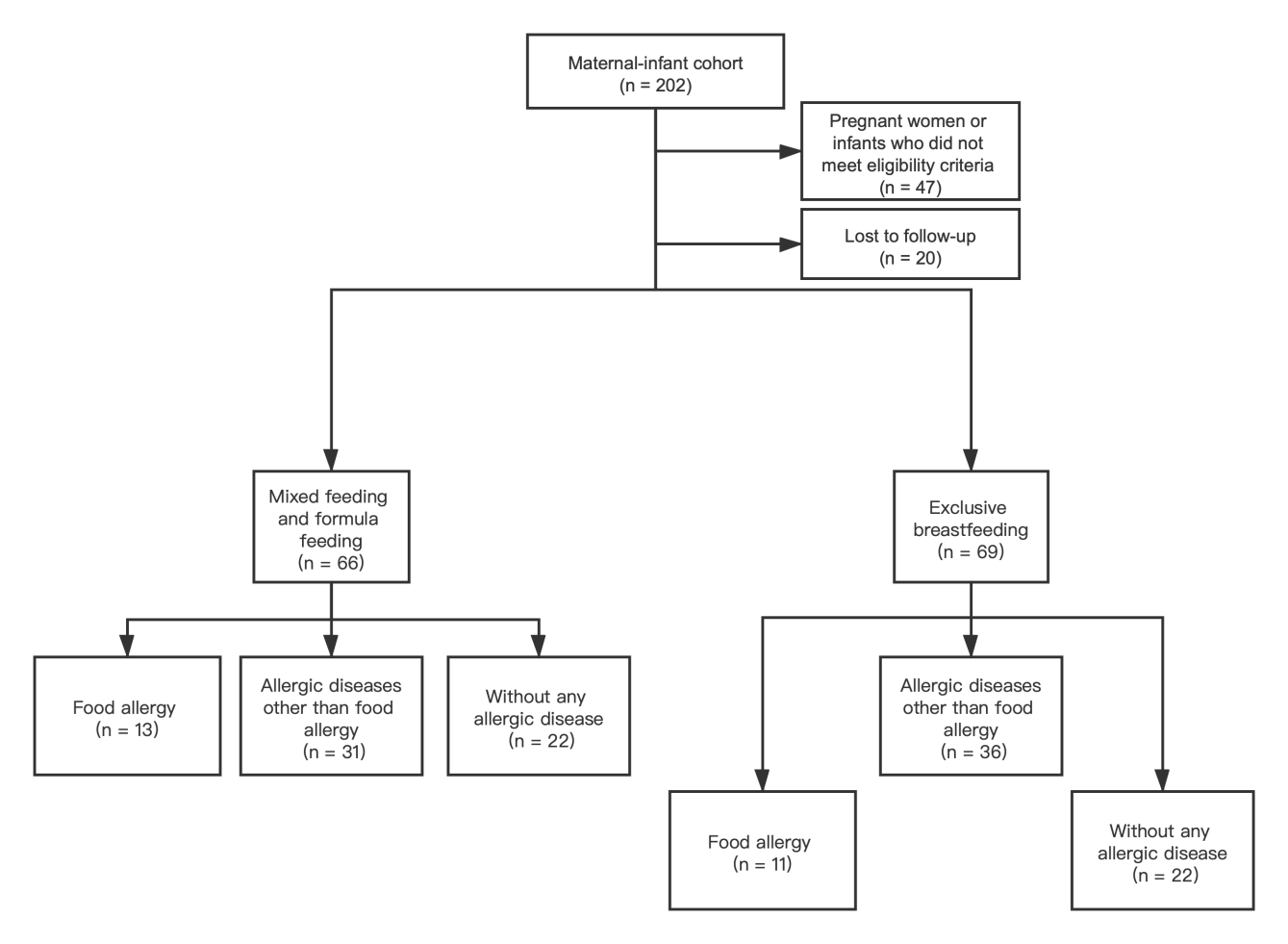


**Supplementary Figure 1.** Flowchart of participant selection in this study.

**1.2 Supplementary Table**

**Supplementary Table 1. Characteristics of food-allergy and non-allergy infant participants in this study**

| **Characteristic** | **Food allergy (n = 24)** | **Non-allergy (n = 44)** | ***P*** |
| --- | --- | --- | --- |
| **Sex** |  | | 0.976 |
| Male, n (%) | 13 (54.2) | 24 (54.5) |  |
| Female, n (%) | 11 (45.8) | 20 (45.5) |  |
| **Mode of delivery** |  | | 0.69 |
| Vaginal, n (%) | 18 (75) | 31 (70.5) |  |
| Cesarean, n (%) | 6 (25) | 13 (29.5) |  |
| **Time for complementary food** |  | | 0.341 |
| <6 mo, n (%) | 11 (45.8) | 15 (34.1) |  |
| >6 mo, n (%) | 13 (54.2) | 29 (65.9) |  |
| **Contact with pets** |  | | 0.196 |
| Yes, n (%) | 7 (29.2) | 7 (15.9) |  |
| No, n (%) | 17 (70.8) | 37 (84.1) |  |
| **Use of antibiotics** |  | | 0.699 |
| Yes, n (%) | 5 (20.8) | 11 (25) |  |
| No, n (%) | 19 (79.2) | 33 (75) |  |
| **Siblings** |  | | 0.821 |
| Yes, n (%) | 7 (29.2) | 14 (31.8) |  |
| No, n (%) | 17 (70.8) | 30 (68.2) |  |
| ***Feeding patterns*** |  | | **0.743** |
| ***Breastfeeding, n (%)*** | ***11 (45.8)*** | ***22 (50)*** |  |
| ***Mixed feeding, n (%)*** | ***13 (54.2)*** | ***22 (50)*** |  |

*P < 0.05 indicates statistically significant difference.*
